# Cholera Infantum*Read before the West Texas Medical Association, June 26, 1902.

**Published:** 1902-09

**Authors:** S. Burg

**Affiliations:** San Antonio, Texas, Late Physician of the Imperial Hospital of Vienna


For Texas Medical Journal.

### Cholera Infantum.*

BY S. BURG, M. D., SAN ANTONIO, TEXAS,
Late Physician of the Imperial Hospital of Vienna.

In speaking of cholera infantum one usually understands all the different forms of diarrhea in children occurring during the hot season, a definition that is scientifically incorrect. Cholera infantum is only one special form of the summer diarrheas in children, and percentually the rarest one.

But taking into consideration the general meaning of the word, I will speak about the different forms of summer diarrhea, as they occur in general practice. The reason why these diseases are so numerous in the hot summer season has been given by authors at different times in a different way. First, atmospheric influences,

*Read before the West Texas Medical Association, June 26, 1902.

such as the various barometrical pressures, air currents, etc., have been considered causative. Others, again, accused telluric influences for being the originators of the disease.

An English author said that whenever the temperature three feet under the surface of the soil goes over fifty degrees then the disease will gain in extension and intension. All these theories have been proven to be futile. One fact has been brought out unanimously by all the authors of modern times, that the high temperature of the atmosphere is the only causative factor, because it furnishes one of the essentials in the development of the germs which invade the alimentary tract; and by their immense multiplication will, according to different aiding moments, cause all these forms of diarrhea, which kill the infants and children by the thousands, and bring many a trying hour to the attending practitioner. The aiding factors, which help the germs to do their deleterious work, are *faulty nutrition* and *unhealthy surroundings.*

The artificially-fed children, especially of the poorer classes, who lack the understanding of cleanliness of person and surroundings, will furnish the largest number of victims of summer complaints. In breast-fed children summer diarrhea is less frequent, and here, too, the faulty nutrition consists in overfeeding the babes, by letting them nurse to overflow. How many mothers apply their babes every time they cry, without considering that a short while ago they had a good meal at the breast. They may be thirsty and cry for water, and still they are given food. If we consider that the high temperature will weaken the children and debilitate the resistency of their nerves, we will understand why the surplus of food cannot be overcome by the digestive organs. It will act as an irritating foreign body and give a good pabulum for the development of the germs, which are always present in the alimentary tract of children. Not only overfeeding at the breast, but menstruation, psychical emotions or pregnancy of the mother, will influence the breast milk in such a way as to act like a poisonous matter on the child.

We have, then, those forms of simple, or functional, diarrhea without vomiting and without elevation of temperature, which are easily overcome. A simple purgative, either a teaspoonful of castor oil or a few doses of one-half grain of calomel, a good irrigation of the bowels with normal salt solution, will soon remove the morbid condition. Milk food must besides be stopped for a short while, or weaning of the child in case of pregnancy of the mother must follow. Mothers cannot be impressed enough with the necessity of feeding their infants less in summer, by allowing larger intervals between meals, and making the rations smaller, and instead give them plenty of water.

If, in addition, frequent bathing would be resorted to and the dressing be less heavy, the number of diseases would be much smaller. Another moment that will add to the gravity of faulty nutrition and which is looked at by a good many as a special cause of diarrhea is *dentition.* The question, whether dentition as such will cause summer diarrhea is not fully answered in the affirmative. Continental authors, especially Germans, like Kassowitz and Widerhofer, are inclined to deny it.

In artifically-fed children, there is always a cause to be found in the nutrition, without regard to dentition. But even in breast-fed children we will, with more careful examination, find that either overfeeding exists, or that the child has been given a little food beside the breast, like a crust of bread, that has been sucked for a long while, until it turned sour; or a piece of meat, or a bone, from which some parts have been torn off and swallowed. All these things have been smeared by the child into nearly everything on the table, on the pillow, or they may have fallen to the ground and been picked up and handed to the child again, not to forget the flies that have beleagered the above mentioned eatables, and which are known today to be the propogators of all kinds of infections. Is it, then, necessary to look for hypothetical causes, like dentition, if there are so many others more tanglible to our command?

I will admit, that in some children dentition may cause a nervous irritability, that the augmented salivation will, if swallowed, lessen the acidity of the stomach. These factors may create in such children a kind of predisposition for catarrhal affection of the digestive tract and cause a shortly lasting functional diarrhea; but to it there must needs come a *materia peccans* from outside, which, according to the foregoing, is always in play to bring about the clinical picture of inflammatory diarrhea. It is necessary, therefore, to emphasize the fact, especially among the laity, not to look at dentition as the only cause for summer complaint and not to take it as a condition that has to be, against which nothing can be done. Many a child has for that reason been neglected and let go on to a condition where there was no help.

The form we meet with usually in bottle-fed children, under the influence of the above mentioned agents of faulty nutrition, and dirty, unhealthy surroundings, is *cholera infantum,* which in its clinical and etiological features resembles the cholera morbus of the adult. The evildoer is that peculiar comma bacillus, which, by the way, according to Prior and Finkler, resembles in all particulars that of the true Asiatic cholera of Koch, and also that ubiquitous *bacterium coli communis,* which, in conjunction with *streptococcus, staphylococcus,* and a good many other bacteria, saprophytic in their nature, are usually found in this as well as in all other acute and chronic mycotic diarrheas of children.

As to the treatment of this severe affection, we are still obliged to adhere to the old known empirical methods as long as the causal micro-organism and respective antitoxin is not found.

At the outset, I use a purgative in the form of the old familiar castor oil or, still better, calomel, which through its purgative and antiseptic qualities has the preference. If vomiting is severe I have the calomel, one-fourth to one-half grain, with a little sugar, rubbed into the tongue and buccal mucosa. An irrigation of the large bowel with common salt solution, or a solution with a mild antiseptic like permanganate of potash, is used in addition, one to two quarts at the time, and repeated twice to three times daily. All food is forbidden, except cold water in small quantities. If vomiting and purging continue, pain is present as shown by the restlessness and crying of the child, the only remedy to be relied upon is a hypodermic injection of one-eightieth to one-tenth of morphia with 1-800 to 1-600 grain of atropia. Time must not be lost in using this only remedy at our command, until the algid stage comes on with those brain symptoms, commonly called hydrencephaloid, in which condition opiates should rather be avoided, as they precipitate the fatal end. In that extreme condition hypodermoclysis —that is, subcutaneous injection of normal salt solution—and the use of musk in suppositories (one to two grains every two or three hours) by rectum is indicated. I prefer musk, as in my mind it is a much superior heart stimulant than strychnia. Cold applications to the abdomen and, in case of high fever, cold packs of the whole body are a good supplement to the medicinal treatment.

If the severity of the condition, especially the vomiting, subsides, nourishment in form of rice water or barley water and light toddies are given, followed by meat broths diluted with water, and only gradually and cautiously milk nourishment is instituted. But if the condition has gone so far that the child is semi-comatose, tossing its head from one side to the other, the fontanellae are sunken, the eyes deep in the cavities with dark rings around them and half open, with ulcera on the cornea, the skin dry, cyanotic, which can be lifted up in a fold that will stay, then hardly any remedy will do, and the fatal end will soon close the scene.

More frequently we meet with *acute and chronic mycotic enteritis* with all its gradations in severity and sequelæ. It is to be considered as an intestinal catarrh of the whole intestinal tract, not excluding the stomach, with either no visible alterations of the mucosa, except a hyperemic condition with swelling, which is hardly apparent in post-mortem examination, or the inflammation has gone so far that the epithelium of the mucosa will be exfoliated and now and then extend in diameter and deep into the follicles and crypts in the form of exulcerations, also visible to the naked eye. The lymphatic glands in the submucosa and in the mesentery will, in the course of time, and in chronic cases, also be affected, and become swollen, sometimes to such an extent that they are palpatable through the thinned abdominal wall. The symptomatology of the *acute form* resembles in the beginning, inasmuch as vomiting and diarrhea open the scene, that of cholera infantum. But if in the latter the acuteness grows pretty quickly, the number of stools being often as high as twenty and more daily, of the color of rice water and of neutral, or alkaline reaction, in acute mycotic enteritis, vomiting will either subside soon or every now and then reappear; the stools will not be so frequent, occasionally from eight to twelve or fifteen daily, mostly of a green, sometimes yellow-brownish, color, watery, containing more or less mucus, also masses of undigested milk in form of curds, fat globules resembling casein, or the stools look like chopped eggs. The smell is always offensive; sometimes sour, sometimes that of foul eggs. The reaction is acid. If the inflammation has lasted for some length of time, in very acute cases, even in the beginning, blood in patches or streaks will be admixed to the mucus.. Just what kind of bacterium is the cause of the respective color of the stool and of the peculiar smell is, unfortunately, not known, just as well as we are in the dark about the question whether this or that bacterium is the cause of this or that form of diarrhea. In nearly all the forms the intestinal contents swarm with the different forms of bacteria, foremost of all the *bacillus coli communis,* the streptococcus and staphylococcus. Just as in the former types our treatment in this form of diarrhea will be groping in the dark, as long as the special bacillus for each special form and its respective antitoxin is not found.

In the meantime our treatment must be a symptomatic one, our aim being to check bacterial development and the poisoning by the toxins, by removing the irritating contents from the bowel and instituting intestinal antisepsis, and, what is of greatest value, in treating the indigestion, by correcting the mode of nourishing the children. If we succeed in the latter point, we will be victorious in the fight against summer diarrhea, as the first indications are easier complied with.

If I have reason to assume that the stomach as well as the bowels contain undigested and fermenting material, and this nearly always is the case, I begin with calomel in one-fourth or one-half grain doses until three to six grains are taken, sometimes, especially if nausea and vomiting are not very excessive, combined with one or two grains of salol. This medication is supported by salt water irrigation of the bowels. If called in the beginning of the disease, I stop all milk nourishment and allow only cool water or rice water; later, if the severity of the symptoms have subsided, and usually it is the case in twenty or thirty-six hours, I allow albumen water, or oatmeal, or barley decoction with sugar and a pinch of salt in it. A good temporary food, especially if a stimulant is desirable, is Jacobi's mixture, consisting of about five ounces of barley water, a teaspoonful of good whiskey or brandy, with sugar and a little salt. As long as the diarrhea is frequent I order irrigations of the bowels once or twice daily, and internally I give one of the known astringents, in combination with opium. I have mostly used to satisfaction resoscin in one-fourth to one-half grain doses with one-third to one-half grain of Dovers' powder every two hours. If fever is frequent I combine it with one-half to one grain of phenacitin. Occasionally bismut. subnitr. or bismut. salicylate was substituted for resorcin, especially if a continued use of the antiseptic and astringent was necessary. As to remedies used in this class of cases, it is needless to say that their number is legion, every now and then a new remedy being praised as a panacea. But good judgment of the physician is required to make his choice to advantage. In general, it will be necessary to employ alkalines, like the chalk mixture, bicarbonate of soda, in cases of sour fermentation; and acids, like muriatic acid, in cases where the stool has a foul odor, both in combination with antiseptics and opiates. In the latter condition creosote, four to six drops, or carbolic acid, two to four drops, in a four-ounce mixture has answered the purpose. When the fever subsides, the stools assume a more natural quality and lose their bad odor, and the child seems to have a desire for food, then milk nourishment is again instituted, but very cautiously. At first I allow one part of good boiled milk, kept in an air-tight bottle on ice, to five or six parts of barley or oatmeal water, and if well borne gradually change in proportions in augmenting the parts of milk. As regards the use of opium in the treatment of summer diarrhea, I think that it is the best remedy at our command. Although I avoid its use in the beginning of treatment, provided there is no great pain and restlessness, I hardly ever omit it in any prescription. As anodynes, bromide of potassium and chloral hydrate may occasionally take its place, but we cannot dispense with opium. Jacobi says: "Opium, by its inhibitory effect on reflexes, diminishes hyperaesthesia, hyperperistalis and hypersecretion. The objections to its use are only theoretical." Often all our efforts to check the trouble will be of no avail, especiallly in those cases where the faulty nutrition and unhealthy surroundings cannot be corrected. Then the case will either go on from bad to worse, and the fatal end will follow under the picture of hyprencephaloid, or the case will become *chronic*. The number of stools wili diminish, but still will be greater than normal; their character will change from day to day, its bad odor persisting, and the mucus being more abundant. With exacerbations and ameliorations exchanging, the disease may go on for weeks and then we may be confronted with that deplorable condition which the French call athrepsia, a real picture of misery. The emaciation is extreme, the skin pale, of an ashy hue, the eyes sunken, the extremities, like little sticks, covered with flabby skin, the face pointed, with an expression of extreme suffering. The final act of the physician is then the writing of a death certificate, with cause given as cholera infantum and as secondary cause inanition.

In the majority of cases fortunately our efforts are crowned with success. Although the diarrhea drags on occasionally for a long time, we are able to correct the condition of the bowels. Despite of occasional relapses the child's appetite becomes better, and it does not seemingly lose much in weight. Often the diarrhea will be followed by constipation. The rare stools look grayish, putty like, lumpy, with an extremely bad odor. These lumps are mostly taken for casein, but consist of undigested fat. In such a condition I have found the natural ferments like pepsin—pancreatin in combination with mur. acid do good, but an occasional dose of castor oil was not forgotten. If the little patient could be sent to a cooler place in the country, and especially if the milk food could be regulated according to physiological requirements, even very obstinate cases of chronic diarrhea terminated in recovery.

The fourth form we meet with begins like acute mycotic enteritis, with vomiting and diarrhea, but after a few days assumes the forms of an *ileo-colitis or dysentery,* with tenesmus and frequent maloderous stools of a greenish, bloody or dark brown color, containing masses of mucus and blood. The anatomical changes of the color are here especially intensive and extensive, forming ulcerations of different forms and sizes, occasionally even diphtheritic in character. The line of treatment is the same as in the previous forms, namely, first eliminations of irritant matter, and, second, the use of intestinal antiseptics. Intestinal irrigations, with antiseptics, especiallly permanganate of potash or a mild solution of nitrate of silver, one-half to one per cent., are very useful. Subnitr. of bismut. and opium are the remedies par excellence; also nitrate of silver, one-fiftieth to one-sixtieth of a grain internally, best given in a decoction of simmaruba. Most effective I have found in this class of cases a teaspoonful of castor oil with eight to fifteen drops of paregoric for several days. The tenesmus subsides and the stool assumes a more natural quality, and in course of a week or ten days the disease is overcome. Some cases with severe streptococcus infection will end fatally. Others, again, assume a chronic character which will finally yield after a tedious treatment on the same line like chronic enteritis.

The treatment of summer diarrhea in its different forms is, therefore, by no means an easy one. Many a time we have consulted our books or journals, or sought the counsel of our confreres in a special case, and for no avail. I cannot but cite in this connection the words of Jacobi, who says: "Never is the common sense and tact of the intelligent practitioner more thoroughly taxed. In regard to that there can be no law. No printed rule ever supplies or substitutes brains."

